# Escaping ESKAPE resistance: *in vitro* and *in silico* studies of multifunctional carbamimidoyl-tethered indoles against antibiotic-resistant bacteria

**DOI:** 10.1098/rsos.230020

**Published:** 2023-04-19

**Authors:** Kanika Gulia, Ahmed H. E. Hassan, Justin R. Lenhard, Abdelbasset A. Farahat

**Affiliations:** ^1^ Master of Pharmaceutical Sciences Program, California Northstate University, 9700 W Taron Dr., Elk Grove, CA 95757, USA; ^2^ College of Medicine, California Northstate University, 9700 W Taron Dr., Elk Grove, CA 95757, USA; ^3^ Department of Medicinal Chemistry, Faculty of Pharmacy, Mansoura University, Mansoura 35516, Egypt; ^4^ Department of Pharmaceutical Organic Chemistry, Faculty of Pharmacy, Mansoura University, Mansoura 35516, Egypt; ^5^ Department of Clinical and Administrative Sciences, College of Pharmacy, California Northstate University, Elk Grove, CA 95757, USA

**Keywords:** indole, antibiotic resistance, antimicrobial agents, multifunctional compounds

## Abstract

Combining the hybridization and repurposing strategies, six compounds from our in-house library and having a designed hybrid structure of MBX-1162, pentamidine and MMV688271 were repurposed as potential antibacterial agents. Among, compounds **1a** and **1d** elicited potential sub-µg ml^−1^ activity against the high-priority antibiotic-resistant Gram-positive members of ESKAPE bacteria as well as antibiotic-susceptible Gram-positive bacteria. Furthermore, they showed potential low µg ml^−1^ activity against the explored critical-priority antibiotic-resistant Gram-negative members of ESKAPE bacteria. In time–kill assay, compound **1a** has effective 0.5 and 0.25 µg ml^−1^ antibacterial lethal concentrations against MRSA in exponential growth phase. *In silico* investigations predicted compounds **1a** and **1d** as inhibitors of the open conformation of undecaprenyl diphosphate synthase involved in bacterial isoprenoid synthesis. In addition, compounds **1a** and **1d** were predicted as inhibitors of NADPH-free but not NADPH-bound form of ketol-acid reductoisomerase and may also serve as potential B-DNA minor groove binders with possible differences in the molecular sequence recognition. Overall, compounds **1a** and **1d** are presented as multifunctional potential antibacterial agents for further development against high- and critical-priority Gram-positive and Gram-negative antibiotic-resistant ESKAPE bacterial pathogens as well as antibiotic-susceptible Gram-positive bacterial pathogens.

## 1. Introduction

A doubtless fact is that microbial diseases including bacterial infections presented serious threats and major challenges to health since the dawn of the history. Several ancient human mummies or remains show infections by pathogenic bacteria such as *Escherichia coli*, *Staphylococcus saprophyticus*, *Brucella melitensis*, *Gardnerella vaginalis*, *Salmonella enterica* and many others [1–3]. Despite the successful development of antibiotics in the twentieth century which enabled effective combating of such bacterial diseases, no new class was ever introduced since the 1980s [4,5]. Unfortunately, the evolvement of antimicrobial resistance (AMR) coupled with the low discovery and introduction rate of novel and clinically effective antibacterial agents over the past decades might be returning us gradually to the pre-antimicrobial era [6]. Doubtless, the attrition of the usefulness of currently available antimicrobials poses heavy burdens and results in a health crisis which is estimated to claim 10 million lives globally by year 2050 [7–9]. Consequently, it is urgent to develop new antimicrobial agents [10].

Based on mortality, burden, resistance, transmissibility, preventability and treatability, WHO Pathogens Priority List Working Group disclosed in 2018 a list of bacteria with critical, high and medium priority for new drug development [11]. The critical priority involved four bacteria: carbapenem-resistant *Acinetobacter baumannii*, carbapenem-resistant *Pseudomonas aeruginosa*, carbapenem-resistant and third-generation cephalosporin-resistant *Klebsiella spp*., and third-generation cephalosporin-resistant *Enterobacter spp*. Two bacteria among the high-priority bacteria: vancomycin-resistant *Enterococcus faecium* and methicillin-resistant *Staphylococcus aureus*, constitute with four critical-priority bacteria the ESKAPE bacterial pathogens which are responsible for most of the life-threatening nosocomial infections [12]. Accordingly, it is highly demanded to swiftly develop new antimicrobial agents combating these highly virulent and multi-drug-resistant ESKAPE pathogens.

ESKAPE pathogens can escape from the action of antimicrobials through inactivating or altering drugs, modification of the drug binding sites, altered permeability to reduce intracellular accumulation, and/or formation of biofilm [13]. Circumventing resistance to existing drugs might be achieved via developing new agents acting on new molecular targets as well as recruitment of polypharmacology by developing multi-target agents that inhibit more than target [14,15]. Although the concept of developing multi-target polypharmacologic agents is well acknowledged in the development of anti-cancer agents [16–20] and anti-inflammatory agents [21], it is yet underexploited for development of antimicrobial agents and limited examples exist.

It is well established that bacterial isoprenoid synthesis pathway differs from the pathway in human and animals [22,23]. The facts that isoprenoid synthesis is dispensable for peptidoglycan synthesis essential for integrity of bacterial cell wall, and, at the same time, bacterial enzymes involved in isoprenoid synthesis are mostly different from those of human and animals suggests targeting bacterial isoprenoid synthesis as a strategy for development of novel antibacterial agents [22,24]. The bacterial enzyme undecaprenyl diphosphate synthase (UPPS) has been reported as a potential target for development of novel antibacterial agents toward inhibition of bacterial isoprenoid synthesis and, hence, impairing bacterial cell wall [22,24]. While there is a human version of farnesyl pyrophosphate synthase (FPPS) homologous to the bacterial FPPS, a bacterial but not human version of UPPS exists. Accordingly, a compound impacting UPPS might possess a desirable selectivity.

DNA replication and gene transcription are both essential processes for bacterial growth and survival. Accordingly, selective targeting of bacterial DNA could be a promising strategy to develop novel effective antibacterial agents. In fact, both processes require promotor sequences as starting sites where DNA would unwind, and the processes would initiate recruiting nucleic acid polymerases and transcriptases. Unlike human DNA in which the most abundant promotor sequences are GC-rich [25], the most abundant promotor sequences in bacteria and parasites are AT-rich [26,27]. Consequently, developing molecules that can recognize and bind to AT-rich repeated sequences might afford potential antimicrobial agents [28–42]. Such a strategy might be augmented by the phenomenon of sequence-dependent DNA shape where hydrogen bonds might be formed between B-DNA and minor groove binding small molecules but not with side chains of many important DNA-interacting proteins [43]. In addition, the minor groove of B-DNA AT-rich sequences are narrower, deeper and more capable of establishing polar interactions than that of GC-rich sequences.

Ketol-acid reductoisomerase (KARI) also known as acetohydroxyacid isomeroreductase (AHIR), is an essential enzyme for synthesis of the three branched chain amino acids (L-leucine, L-isoleucine and L-valine) which are important for bacterial survival [44]. While it exists in bacteria, fungi and plants, there is no human or animal version of KARI [45]. Development of a KARI inhibitor would allow targeting bacterial synthesis of branched chain amino acids and would produce selective antibacterial effects. Consequently, it is an attractive strategy for developing novel and safe antimicrobial agents [46].

## 2. Results and discussion

### 2.1. Repurposing rational

Repurposing (aka repositioning or reprofiling) is a well-acknowledged strategy in drug discovery that offers several advantages including saving time, money and resources that are needed to develop novel drugs from scratch [47–52]. This strategy is not limited to repurposing compounds for indications outside of clinically approved therapeutic areas, but extends also to previously studied molecules that have not been approved or even previously failed [19,53]. Several molecules were successfully rediscovered for novel clinical uses by utilizing such a strategy. In lieu of the urgent need to achieve novel bacterial agents to overcome the ever-increasing threats of bacterial multi-drug resistance (MDR), repurposing might be a good choice.

**Figure 1.**
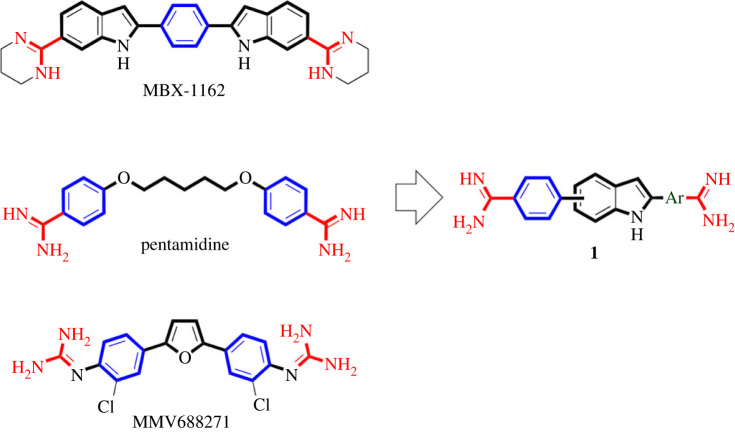
Reported antibacterial starting points for the design and rational repurposing of targeted compounds as multifunctional novel antibacterial agents.

As above-mentioned, multifunctional molecules modulating multi-targets can help to overcome MDR. This approach might be augmented through inclusion of novel molecular targets among the addressed multi-targets. Therefore, a multifunctional molecule modulating the novel antibacterial molecular targets, UPPS and ketol-acid, as well as inhibiting DNA replication and gene transcription through binding to minor groove of bacterial AT-rich DNA sequences might be a promising antibacterial agent against MDR. Recently, MBX-1162 (figure 1), a 1,4-bis(1*H*-indol-2-yl)benzene scaffold bearing diamidine moieties, was discovered as potential lead inhibitor of UPPS of *S. aureus and E. coli* for development of novel antibacterial agents [24]. Interestingly, MBX-1162 was also found to be a minor groove binder targeting bacterial DNA [54,55]. Meanwhile, pentamidine (figure 1), an antiparasitic drug possessing 1,5-bisphenoxypentane scaffold diamidine moieties, is a known DNA minor groove binder with reported antibacterial activity [56]. In addition, pentamidine was found to inhibit KARI of *E. coli* [57]. Compound MMV688271, a 2,5-bisphenylfuran bearing diguanide moieties that has structural similarity to the diamidine, is another interesting compound that inhibits KARI of *Mycobacterium tuberculosis* [58]. Considering compounds MBX-1162, pentamidine and MMV688271, they share common structural features involving: (i) a central flat aromatic ring (phenyl or furan for MBX-1162 and MMV688271, respectively) or alkyl chain (for pentamidine), (ii) two flat aromatic rings (indole rings for MBX-1162 or phenyl rings for pentamidine and MMV688271) attached to the right and left of the central moiety, and (iii) two amidine or guanidine moieties attached to the right and aromatic rings.

**Figure 2.**
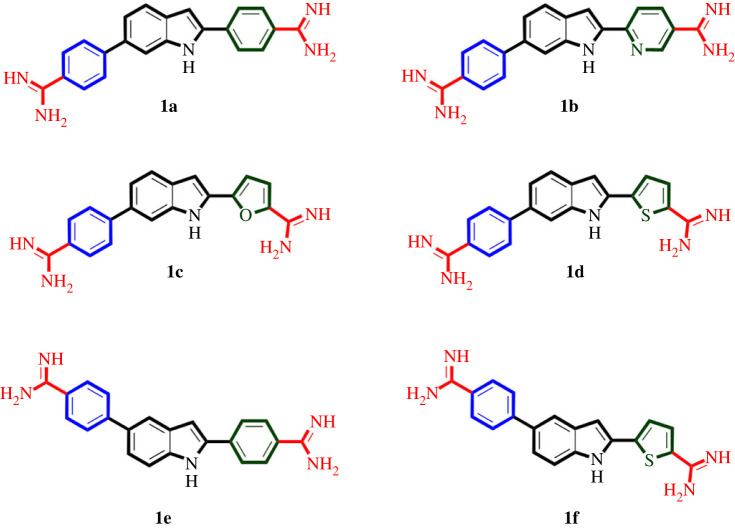
Structures of selected compounds for repurposing as antibacterial agents against ESKAPE.

As molecular hybridization is a powerful approach in attaining multifunctional molecules [16,19,21,59,60], we thought a hybrid molecule **1** (figure 1) of MBX-1162, pentamidine and MMV688271 might target bacterial UPPS, KARI and DNA to elicit potential antibacterial activity against MDR ESKAPE pathogenic bacteria. The structure of such hybrid molecule **1** might maintain the left phenyl ring of pentamidine and MMV688271, while it incorporates the aromatic indol-2-yl moiety of MBX-1162 but as a central feature instead of phenyl, furan or alkyl chain of MBX-1162, MMV688271 and pentamidine, respectively. The maintained phenyl ring might be coupled to 5- or 6-position of the central indole moiety while 2-position would be coupled to variable aromatic rings as a replacement to the right phenyl ring of pentamidine and MMV688271 and the right indole ring of MBX-1162. Finally, the two diamidine moieties would be placed on each of the introduced right aromatic moieties and the left phenyl moiety. Searching our available in-house compounds library, we have selected six compounds (figure 2) conforming to the proposed structure **1** (figure 1) that were originally prepared as antiparasitic agents and took the mission of exploring possible repurposing of these compounds as multifunctional antibacterial agents to combat pathogenic ESKAPE bacteria [61].

### 2.2. Biological evaluations

#### 2.2.1. Evaluation of spectrum and minimum inhibitory concentrations against ESKAPE

To get insights into the activity spectrum of the compounds, a panel of bacterial pathogens was used to assess minimum inhibitory concentrations (MICs) and activity spectrum which involved two Gram-positive and two Gram-negative MDR ESKAPE pathogenic bacterial strains, as well as two Gram-positive and three Gram-negative antibiotic-susceptible pathogenic bacterial strains (table 1). Briefly, the used Gram-positive antibiotic-resistant strains of ESKAPE involved: (i) the vancomycin-resistant *E. faecium* (VRE; Antibiotic Resistance Isolate Bank #0572), which is resistant to vancomycin, penicillin, ampicillin, doxycycline, levofloxacin, rifampin, teicoplanin and quinupristin/dalfopristin combination; (ii) the methicillin-resistant *S. aureus* (MRSA; subsp. *aureus* COL), which is resistant to methicillin, cefoxitin, linezolid, levofloxacin, erythromycin, penicillin and oxacillin. Meanwhile, the used Gram-negative antibiotic-resistant strains of ESKAPE involved: (i) the carbapenem-resistant *Enterobacterales* (CRE); *K. pneumonia* (Clinical Isolate #015), which is resistant to ampicillin, ampicillin/sulbactam combination, aztreonam, cefazolin, cefepime, cefotaxime, cefoxitin, ceftazidime, ceftriaxone, ciprofloxacin, doripenem, ertapenem, imipenem, levofloxacin, meropenem, piperacillin/tazobactam combination, tetracycline, tobramycin and trimethoprim/sulfamethoxazole combination; (ii) the carbapenem-resistant *A. baumannii* (CRAB; Clinical Isolate no. 03-149.2) which is resistant to meropenem, polymyxin and ampicillin/sulbactam combination. On the other side, the used antibiotic-susceptible strains involved *E. faecalis* (Antibiotic Resistance Isolate Bank no. 0671) and methicillin-susceptible *S. aureus* (MSSA ATCC 25923) as Gram-positive bacterial strains, in addition to *A. baumannii* (ATCC 19606), *P. aeruginosa* (Antibiotic Resistance Isolate Bank no. 0238) and *E. coli* (Antibiotic Resistance Isolate Bank no. 0017) as Gram-negative bacterial strains. For comparison, the drugs vancomycin and gentamicin were used as reference standards for Gram-positive and Gram-negative bacteria, respectively. The interesting outcome of evaluation of MICs is included in table 1.

**Table 1.** Determined minimum inhibitory concentrations (µg ml^−1^) for tested compounds and reference standard drugs against antibiotic-resistant Gram-positive and Gram-negative members of ESKAPE in addition to antibiotic-susceptible Gram-positive and Gram-negative pathogenic bacterial strains. G+ve = Gram-positive. G-ve = Gram-negative. VRE = vancomycin-resistant *Enterococcus faecium*. MRSA = methicillin-resistant *Staphylococcus aureus*. MSSA = methicillin-susceptible *Staphylococcus aureus*. CRE = carbapenem-resistant *Enterobacteriaceae* (*Klebsiella pneumonia*). CRAB = carbapenem-resistant *Acinetobacter baumannii*.

compound	antibiotic-resistant G+ve		antibiotic-susceptible G+ve		antibiotic-resistant G-ve		antibiotic-susceptible G-ve		
	*E. faecium* (VRE)	*S. aureus* (MRSA)	*E. faecalis*	*S. aureus* (MSSA)	*K. pneumonia* (CRE)	*A. baumannii* (CRAB)	*A. baumannii*	*P. aeruginosa*	*E. coli*
**1a**	0.25	<0.0625	0.5	0.25	8	1	16	8–16	1
**1b**	4	1	2	1	32	4	32	32	8
**1c**	8	4	32	16	>128	64	32	16	16
**1d**	0.25	0.25	0.25	0.5	16	8	32	16	1
**1e**	16	1	16	1	>128	32	16	32	8
**1f**	16	16	>128	16	>128	>128	64	64	32
vancomycin	8	1-2	2	2	—	—	—	—	—
gentamicin	—	—	—	—	64	>128	8	2	0.5

As expected, MIC value for vancomycin, the used reference drug against Gram-positive bacteria, was relatively high against the antibiotic-resistant VRE, while it inhibited the growth of the used MRSA and antibiotic-susceptible strains at low concentrations (1–2 µg ml^−1^, table 1). As also expected, MIC values for gentamicin, the used reference drug against Gram-negative bacteria, were very high against antibiotic-resistant strains, CRE and CRAB, while it was effective against antibiotic-susceptible *E. coli* and *P. aeruginosa* but less active against *A. baumannii*. However, the CRAB isolate displayed a much higher level of gentamicin resistance in comparison with the carbapenem-susceptible *A. baumannii* (greater than 128 versus 8 µg ml^−1^, respectively, table 1). Interestingly, compound **1a**, which features a phenyl moiety as the right aromatic ring attached to the central indole ring at 2-position, while the left aromatic phenyl ring is attached to 6-position, showed excellent activity against all tested antibiotic-resistant and antibiotic-susceptible Gram-positive bacteria. It was found that all MICs of compound **1a** against VRE, MSSA and *E. faecalis* were in sub-µg ml^−1^ range and were even very potent against MRSA, eliciting an MIC of 62.5 ng ml^−1^ (table 1). In addition, compound **1a** was potentially active against antibiotic-resistant Gram-negative bacteria CRE and CRAB showing single digit µg ml^−1^ MIC values. But in comparison, it showed lower activity against tested antibiotic-susceptible Gram-negative bacterial strains except for *E. coli*. Interestingly, comparing activity of compound **1a** against antibiotic-resistant (CRAB) versus antibiotic-susceptible strains of Gram-negative *A. baumannii,* as well as antibiotic-resistant (MRSA) versus antibiotic-susceptible (MSSA) strains of *S. aureus*, reveals that compound **1a** is more potentially active against antibiotic-resistant strains in both cases. Compound **1b**, which has a single structure difference relative to compound **1a** by possessing the six-membered heterocyclic pyridyl moiety as a replacement of the right phenyl ring at 2-position of the central indole, showed a potentially better activity than vancomycin against antibiotic-resistant Gram-positive bacteria, comparable activity to vancomycin against antibiotic-resistant Gram-positive bacteria, and much better activity relative to gentamicin against antibiotic-resistant Gram-negative bacteria. It is noticeable that compound **1b** showed a similar trend of activity as that of compound **1a** against antibiotic-resistant and antibiotic-susceptible Gram-positive and Gram-negative bacteria. Nevertheless, it was less active than compound **1a**, indicating that introduction of the more polar nitrogen atom might be unfavourable. Meanwhile, compound **1c**, possessing similar structure to compounds **1a** and **1b** but having the five-membered heterocyclic furan moiety as a replacement of the right aromatic ring at 2-position of the central indole, showed further decrease in activity but still showing considerable activity against antibiotic-resistant rather antibiotic-susceptible Gram-positive bacteria. Nevertheless, it lost potential activity against both antibiotic-resistant and antibiotic-susceptible Gram-negative bacteria. Interestingly, the presence of the five-membered heterocyclic thiophene instead of the furan moiety in compound **1d** resulted in restoring the potential activity against antibiotic-resistant and antibiotic-susceptible Gram-positive bacterial strain with MIC values switching again to sub-µg ml^−1^ range (table 1). This could arise from the lower electronegativity of sulphur relative to oxygen which results in lower polarity. The potential activity against Gram-negative bacteria was also restored in compound **1d** relative to compound **1c** but was less than potential compound **1a**. Next, to identification that phenyl and thiophene as right aromatic ring at 2-position of indole afford potential compounds **1a** and **1d**, compounds **1e** and **1f** maintain these moieties, respectively, but differ structurally by having the left phenyl ring attached to 5- instead of 6-position of the central indole ring, which translocates the indole nitrogen atom from the concave to the convex edge of sickle shape of the molecule, resulting in substantial attrition of activity. This indicates that the presence of indole nitrogen in the convex edge of the molecule might result in less favourable interactions or prevent formation of favourable interactions that might be established if present in the concave edge. Together, these findings suggest compound **1a** as potential antibacterial agent against antibiotic-resistant bacteria, especially against vancomycin-resistant *E. faecium* and methicillin-resistant *S. aureus* the high-priority bacteria; both are high-priority pathogens as disclosed by the ‘WHO Pathogens Priority List’.

#### 2.2.2. Time–kill dynamics of compound **1a**

**Figure 3.**
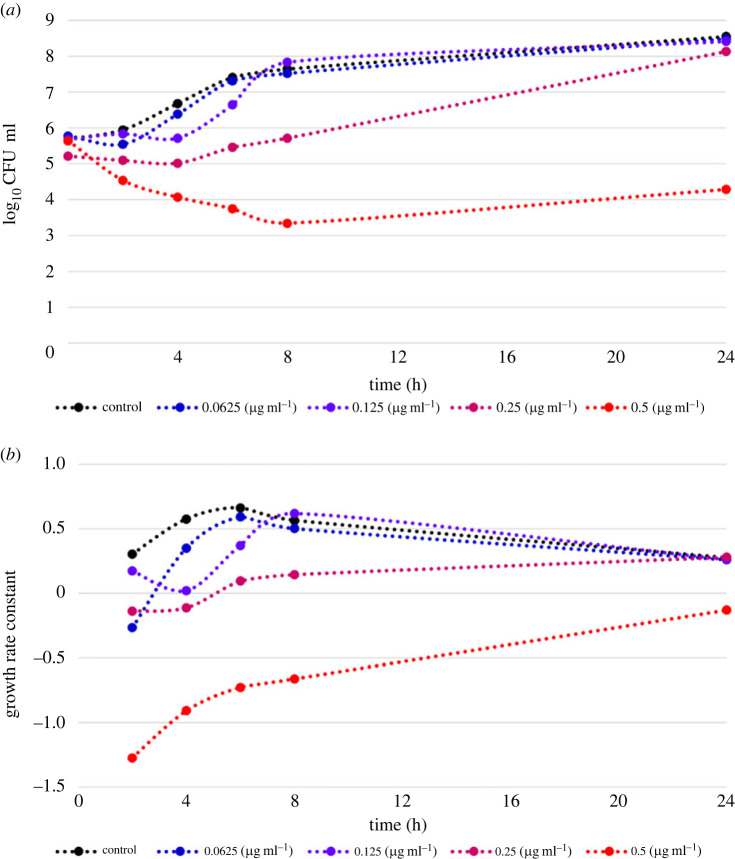
Dynamics of compound **1a** against MRSA over 24 h. (*a*) Time–kill curve for variable concentrations of compound **1a** against MRSA over 24 h. (*b*) Calculated growth rate constants for variable concentrations of **1a** against MRSA over 24 h.

Following the identification of compound **1a** as a potential antibacterial agent, especially against antibiotic-resistant Gram-positive bacteria, eliciting nanogram m^−1^ inhibitory concentration against the high-priority MRSA, it was interesting to explore its rate and extent of bacterial killing against MRSA. Time–kill experiments provide critical insight into the pharmacodynamic effects of potential antibacterials [62]. Accordingly, time–kill curves of MRSA over 24 h using variable sub-µg ml^−1^ concentrations of compound **1a** were explored to interrogate killing and growth of MRSA as a function of both time and concentration (figure 3*a*). In addition, the first-order growth rate constants were calculated and plotted versus time for each used concentration and in absence of compound **1a** (figure 3*b*). The results showed that the untreated MRSA was growing in the exponential-growth phase (log phase) over the first 6 h and maximum growth rate was reached. After 6 h, the growth rate constant started to decline but remained positive suggesting transition of MRSA from exponential-growth phase to enter into stationary phase. Given the information that antibiotic-susceptibility might be different in slowly dividing cells in stationary phase from rapidly dividing cells in exponential-growth phase coupled with the fact that most antibiotics are effective only at exponential growth phase [63], it would be important to understand which growth phase compound **1a** would be effective against. As revealed from results, the calculated first-order growth rate constants for 0.5 µg ml^−^^1^ concentration of compound **1a** were negative over the whole 24 h, and the number of living organisms (colony-forming units) was still lower relative to the initial number, indicating lethal antibacterial activity. However, the slope of the calculated first-order growth rate constants was larger over the period of exponential-growth phase (up to 6 h) than that of the stationary phase. This might suggest that compound **1a**, like most antibiotics, is more effective against rapidly growing bacteria. Nevertheless, the calculated negative first-order growth rate constant after 24 h indicates that compound **1a** at 0.5 µg ml^−1^ concentration is still effective against MRSA in stationary phase. The lower 0.25 µg ml^−1^ concentration of compound **1a** also triggered antibacterial lethal effects up to 4 h confirmed by the calculated negative first-order growth rate constant. Meanwhile the 0.125 µg ml^−1^ concentration elicited bacteriostatic effects at 4 h post treatment with almost zero calculated first-order growth rate constant. Despite the 62.5 ng ml^−1^ concentration of compound **1a**, it showed initial lethal antibacterial activity in the first two hours, and its activity decreased rapidly overtime and started to approach the negative control. These findings indicate that compound **1a** demonstrates more efficacy against MRSA in exponential-growth phase rather than the stationary phase with effective antibacterial lethal concentrations of 0.5 and 0.25 µg ml^−1^. Thus, compound **1a** might be a potential candidate for further development.

### 2.3. *In silico* studies

As compounds **1a** and **1d** were identified as potential antibacterial agents, it was interesting to probe their anticipated possible multifunctional activity. Accordingly, a computational study was initiated to explore whether compounds **1a** and **1d** would be able to dock into UPPS, KARI and DNA as possible biological targets, and what are their possible binding modes and interactions. The interesting outcome is presented in the following sections.

#### 2.3.1. Molecular docking simulation of compounds **1a** and **1d** with UPPS

**Figure 4.**
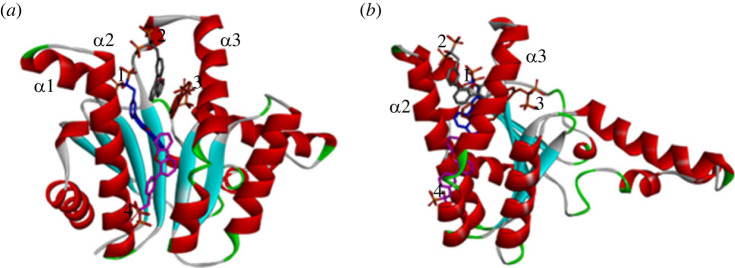
Crystal structure of the open conformation of UPPS accommodating four molecules of bisphosphonate inhibitor coloured in different colours per each molecule (blue, grey, orange and pink) at the four binding sites (PDB code 2E98).

Reported crystal structure of bacterial UPPS has shown to possess four potential binding sites (1–4) (figure 4); any of them can be the binding site of a potential UPPS inhibitor [24,64]. If enough concentration exists, four molecules of some bisphosphonate inhibitors were found to co-crystallize binding to all of these four binding sites (PDB code 2E98; figure 4) [64]. Furthermore, UPPS can adopt either an open or a closed conformation (in regard to *α*2 and *α*3 helices, figure 4*b*), which enables substrates to bind and products release [24,65,66]. Accordingly, *in silico* molecular docking simulation was addressed employing the open and the closed UPPS conformations (PDB codes: 2E98 and 1X06, respectively) to probe whether compounds **1a** and **1d** could dock into UPPS and, if so, to which binding site/conformer.

**Figure 5.**
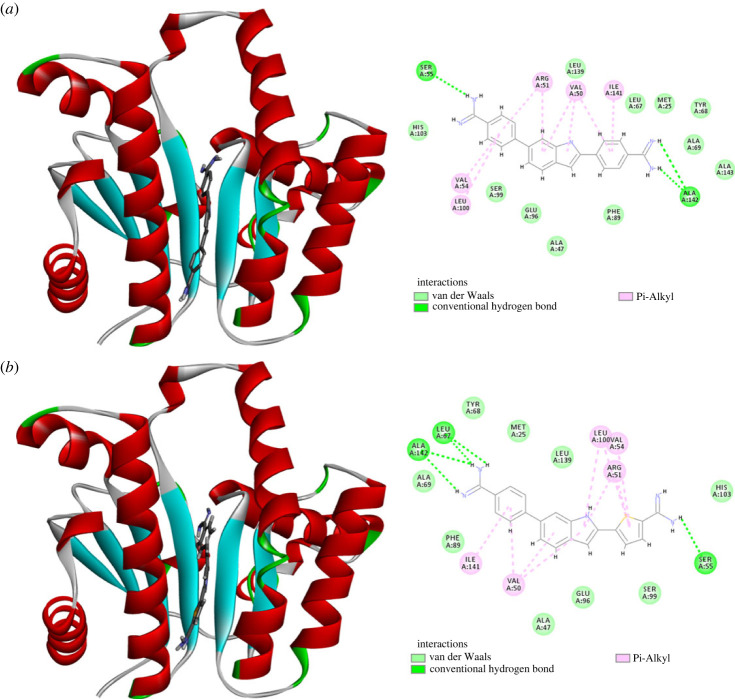
Predicted binding of compounds **1a** and **1d** to the open conformation of UPPS (PDB code 2E98): (*a*) compound **1a** docked at binding site 4 and residues interacting with it; (*b*) compound **1d** docked at binding site 4 and residues interacting with it.

As shown in figure 5, the calculated results suggest that both compounds **1a** and **1d** docked to the open conformation at binding site 4 with calculated favourable binding score of −7.05078 and −6.91088 kcal mol^−1^, respectively (figure 5). Interestingly, none of the retrieved docked poses for both compounds contained predicted binding to any of the other sites 1–3. The calculated best docking pose for compound **1a** showed that its 4-carbamimidoylphenyl moiety at position 6 of the indole ring was placed in binding site 4, while the 4-carbamimidoylphenyl moiety at position 2 was directed towards the β-sheets. However, compound **1d** accommodated the opposite direction placing its 5-carbamimidoylthienyl moiety at position 2 in binding site 4, while the 4-carbamimidoylphenyl moiety at position 6 was directed toward the β-sheets motif. The amidine moieties in both compounds established hydrogen bonds with Ser55 in binding site 4 and with Ala142 in the β-sheets motif. In addition, the central and the peripheral aromatic rings of both compounds established a network of favourable hydrophobic Van der Waal and π-alkyl interactions which was lower for compound **1d**. However, this was partially compensated for by the formed additional hydrogen bond of the amidine moiety of compound **1d** with Leu67 in the β-sheets motif. By contrast to the open conformation, attempted docking of both compounds **1a** and **1d** to the closed conformation did not afford any pose in which the docked compounds can bind with any crucial residue of the binding sites 1–4. Accordingly, it might be deduced that compounds **1a** and **1d** might bind exclusively to the open conformation of UPPS at binding site 4.

#### 2.3.2. Molecular docking simulation of compounds **1a** and **1d** with KARI

**Figure 6.**
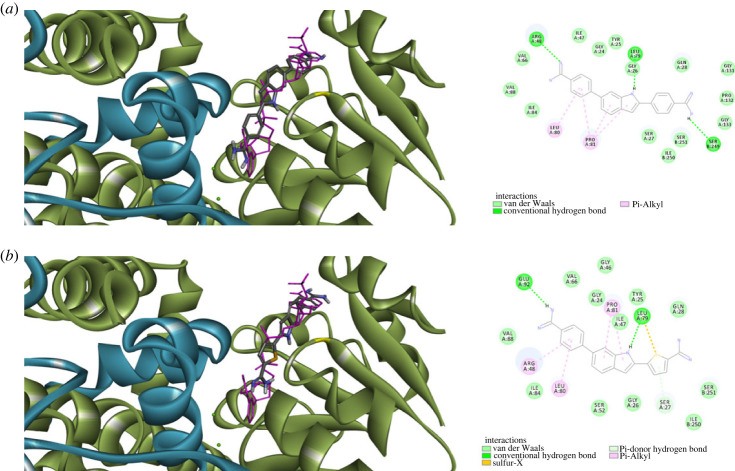
Predicted binding of compounds **1a** and **1d** to dimeric NADPH-free KARI (green: first monomeric chain; blue: interwind C domain of second monomeric chain; pink: NADPH): (*a*) predicted binding mode and interactions of compound **1a**; (*b*) predicted binding mode and interactions of compound **1d**.

Most of bacterial KARI belongs to class I, which has a chain involving less amino acid residues relative to plant KARI, which belongs to class II that possess a chain with almost double amino residues [67]. Consequently, the functional unit of class I bacterial KARI consists of at least a dimer of two chains with the active site formed by intertwined C domains of two chains [67]. In addition, two magnesium atoms and a NADPH cofactor are essential for function of KARI. The structure of enzyme binding only magnesium possesses solvent accessible open active site, which becomes more compact upon binding of NADPH. Based on these two distinct structural features, two types of KARI inhibitors might exist; inhibitors of NADPH-bound or KARI and inhibitors of NADPH-free KARI [68]. Considering these issues, *in silico* molecular docking simulation was performed to assess whether compounds **1a** and **1d** could dock into NADPH-bound or NADPH-free bacterial KARI. Accordingly, molecular docking to active site formed by dimeric KARI structure (PDB code: 7kh7) in presence and in absence of NADPH. As figure 6 illustrates, compounds **1a** and **1d** were able to dock into NADPH-free but not NADPH-bound KARI with calculated favourable scores of −6.70963 and −6.59803 kcal mol^−1^, respectively. Both compounds were bound to NADPH binding site (figure 6) suggesting them to act as competitive inhibitors of NADPH binding KARI and consequently, act as KARI inhibitors. The peripheral carbamimidoylaryl moiety at position 2 of the indole ring in both compounds **1a** and **1d** predicted binding modes were directed towards the intertwined C domains of the second chain, establishing hydrophobic interactions in addition to a hydrogen bond between Ser249 the amidine moiety of compound **1a**. However, most interactions of aryl rings at position 2 of the indole ring of compounds **1a** and **1d**, as well as the central indole and the carbamimidoylaryl moiety at position 6, were contributed by first monomeric chain that involved hydrogen bonds with the amidine moieties and with nitrogen of the indole, as well as several hydrophobic interactions (figure 6). Considering these intricate favourable interactions network of compounds **1a** and **1d** with NADPH-free KARI at the NADPH binding site, coupled with their inability to dock into NADPH-bound KARI, suggests these compounds as possible inhibitors of KARI binding to the NADPH-free but not NADPH-bound form.

#### 2.3.3. Molecular docking simulation of compounds **1a** and **1d** with *S. aureus* DNA

**Figure 7.**
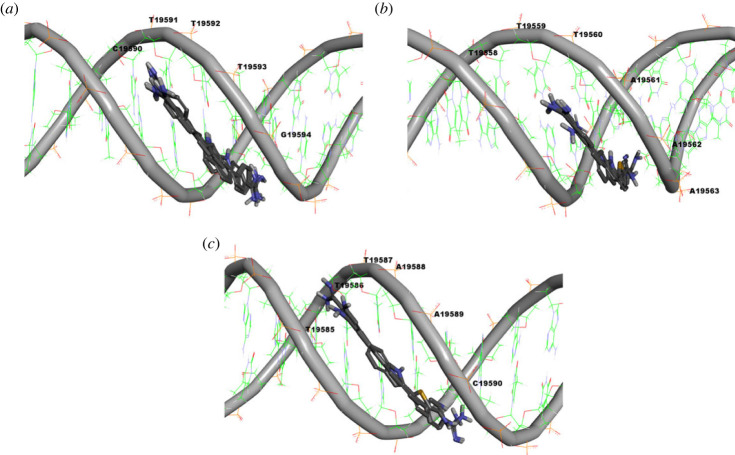
Predicted DNA binding sequences to compounds **1a** and **1d** over sequence 19540–19644 of *S. aureus* TY4, ETB plasmid DNA (GenBank accession no. AP003088): (*a*) predicted binding poses of compound **1a** to CTTTG sequence 19590–19594; (*b*) predicted binding poses of compound **1d** binding to TTTAAAC sequence 19558–19563; (*c*) predicted binding poses of compound **1d** binding to TTAAC sequence 19585–19590.

An *in silico* molecular docking study of compounds **1a** and **1d** interactions with bacterial DNA was addressed to probe for possible presence of a selective molecular recognition process. Thus, compounds **1a** and **1d** were docked to a 105-mer sequence of *S. aureus* TY4, ETB plasmid DNA (GenBank accession no. AP003088, sequence 19540–19644) which involve two sequence repeats. Towards prediction of possible sites for binding, blind docking was initially conducted over the whole sequence of the 105 pair bases. Next, a second docking simulation run was conducted over sequences identified as possible sites to accommodate at least one of compounds **1a** or **1b**. While both compounds were predicted not to bind minor groove of DNA in A-conformation form, the results showed their possible minor groove binding ability to DNA in B-conformation form with predicted binding score ranges of −8.12662 to −8.02435 and −8.09707 to −8.02777 kcal mol^−1^ over the best 10 poses of compounds **1a** and **1b**, respectively. Among the best 10 poses, compound **1a** was bound in six poses to CTTTG sequence 19590–19594 (figure 7*a*), while it showed only one pose for each of the sequences TAAAT 19634–19638, CATTA 19622–19626, TAAAC 19560–19564 and GTATT 19551–19555. On the other side, compound **1d** was bound in five poses to TTTAAAC sequence 19558–19563 (figure 7*b*), bound in four poses to TTAAC sequence 19585–19590 (figure 7*c*), and only one pose for TTATA sequence 19624–19628. These results suggest that both compounds **1a** and **1d** could be potential DNA minor groove binder, and over the studied DNA 19540–19644 sequence, compound **1a** might bind mainly to CTTTG sequence 19590–19594, while compound **1d** might bind mainly to sequences TTTAAAC 19558–19563 and TTAAC 19585–19590. This might reflect the presence of a significant difference in the molecular sequence recognition of B-DNA by compounds **1a** and **1d** and, hence, their elicited biological activity.

## 3. Conclusion

In summary, novel hybrid compounds displayed excellent *in vitro* activity against clinically relevant Gram-positive and Gram-negative bacteria, including high-priority pathogens MRSA, VRE, CRE and CRAB. Out of the tested compounds, compounds **1a** and **1d** showed excellent activity at sub-µg ml^−1^ range against the high-priority antibiotic-resistant Gram-positive members of ESKAPE bacteria as well as antibiotic-susceptible Gram-positive bacteria. In addition, compounds **1a** and **1d** demonstrated potential activity at low µg ml^−1^ range against the used critical-priority antibiotic-resistant Gram-negative members of ESKAPE bacteria but, interestingly, were less active against antibiotic-susceptible Gram-negative bacteria. Studying the time–kill dynamics of the most prominently active compound **1a** against MRSA suggested potential activity against MRSA in exponential growth phase rather than the stationary phase with effective antibacterial lethal concentrations of 0.5 and 0.25 µg ml^−1^. *In silico* investigations of possible interactions of compounds **1a** and **1d** with UPPS as one of the possible molecular targets suggested they might bind exclusively to the open conformation of UPPS at binding site 4 but not to other sites nor the closed conformation. In addition, *in silico* study of possible interactions of compounds **1a** and **1d** with NADPH-free and NADPH-bound KARI suggested both compounds as inhibitors of NADPH-free form of KARI. Furthermore, docking simulation study of compounds **1a** and **1d** over 19540–19644 sequence of *S. aureus* TY4, ETB plasmid DNA demonstrated both compounds as potential B-DNA minor groove binder but revealed differences in the molecular sequence recognition of B-DNA by compounds **1a** and **1d** which might result in different biological responses. Collectively, these findings present compounds **1a** and **1d** as potential agents for further development against high- and critical-priority Gram-positive and Gram-negative antibiotic-resistant ESKAPE bacterial pathogens as well as antibiotic-susceptible Gram-positive bacterial pathogens.

## 4. Experimental

### 4.1. Susceptibility testing

The minimum inhibitory concentrations (MIC) of each study compound were determined against the investigated pathogens using broth microdilution as recommended by the Clinical and Laboratory Standards Institute [69]. In brief, 96-well trays with a maximum volume of 0.5 ml per well were used for susceptibility testing. On the day of each experiment, an overnight culture of bacteria was used to create a turbidity-adjusted concentration of organism suspended in calcium- and magnesium-adjusted Mueller–Hinton broth. Antibacterial stock solutions purchased from AK Scientific were created on the day of each experiment, and a series of two-fold dilutions were used to create a concentration gradient in rows of the tray. The initial inoculum of bacteria in each well was approximately 5 × 10^5^ CFU ml^−1^ suspended in 150 µl of total volume. Completed trays were covered in tape to prevent desiccation, and visible growth was recorded after 18–24 h of incubation at 37°C. Study compounds were evaluated along with vancomycin or gentamicin to ensure consistency with other laboratories. All experiments were completed in quadruplicate. If the results of four experiments were evenly split between two MIC values, then the MIC of the compound was reported as a range between the two values. Compounds were used as dihydrochloride salts and the molecular formula of the salt was used to calculate concentrations.

### 4.2. Time–kill experiments

Time–kill experiments were conducted following a methodology that was described previously [70]. Briefly, overnight cultures of bacteria were used to achieve a starting inoculum of approximately 1 × 10^6^ CFU ml^−1^ of organism investigated in a 50 ml conical tube. Calcium- and magnesium-adjusted Mueller–Hinton broth was used as the nutrient source for the bacteria, and stock solutions of study compounds were prepared on the day of each experiment to achieve 1×, 2×, 4× and 8× the MIC of the study compound in 20 ml of total suspension. The conical tubes were incubated at 37°C with constant shaking, and 100 µl samples were collected from the reaction vessels at 0, 2, 4, 6, 8 and 24 h, serially diluted in saline, and plated onto Mueller–Hinton agar for viable cell counting. Colonies were enumerated following 24 h of incubation at 37°C.

### 4.3. *In silico* simulation studies

*In silico* simulation studies were performed according to known protocols [71,72] using the known X-ray crystal structures of open and closed UPPS conformations (PDB codes: 2E98 and 1X06, respectively), dimeric KARI structure (PDB code: 7kh7), and known sequence of *S. aureus* TY4, ETB plasmid DNA (GenBank accession no. AP003088, sequence 19540–19644), in presence and in absence of NADPH.

#### 4.3.1. For docking study with UPPS

X-ray structure of open and closed conformations (PDB codes: 2E98 and 1X06, respectively) were downloaded from protein data bank as PDB file format and prepared for the docking. Protein preparation steps included deleting all solvent molecules and non-protein molecules and ions, adding hydrogen atoms and calculating charges to standard residues using AMBER ff14SB forcefield. The prepared ligands were docked to binding sites, and the retrieved best poses were visualized and analysed.

#### 4.3.2. For docking study with KARI

X-ray structure of dimeric KARI structure (PDB code: 7kh7) was downloaded from protein data bank as PDB file format and prepared for the docking as described in §4.3.1 except for retaining NADPH molecule. Two docking runs to the binding site were conducted; one run in the presence of NADPH and the other run after deleting NADPH. The retrieved best docked poses were visualized and analysed.

#### 4.3.3. For docking study with *S. aureus* DNA

An *in silico* molecular docking study of compounds **1a** and **1d** interactions with bacterial DNA was addressed to probe for possible presence of a selective molecular recognition process. Thus, compounds **1a** and **1d** were docked to a 105-mer sequence of *S. aureus* TY4, ETB plasmid DNA (GenBank accession no. AP003088, sequence 19540–19644) was downloaded from GenBank and converted into PDB format using BIOVIA Discovery Studio Visualizer. An initial blind docking run was conducted over the whole 105 pair bases sequence followed by second run of docking to sequences identified as possible sites to accommodate at least one of the employed ligands. The retrieved results were visualized and analysed.

## Data Availability

Data associated related to this work was deposited at the Dryad Digital Repository: https://doi.org/10.5061/dryad.c2fqz61d1 [73].

## References

[1] Spyrou MA, Bos KI, Herbig A, Krause J. 2019 Ancient pathogen genomics as an emerging tool for infectious disease research. Nat. Rev. Genet. **20**, 323-340. (10.1038/s41576-019-0119-1) 30953039 PMC7097038

[2] Zink AR, Reischl U, Wolf H, Nerlich AG. 2002 Molecular analysis of ancient microbial infections. FEMS Microbiol. Lett. **213**, 141-147. (10.1111/j.1574-6968.2002.tb11298.x) 12167530

[3] Fricker EJ, Spigelman M, Fricker CR. 1997 The detection of *Escherichia coli* DNA in the ancient remains of Lindow Man using the polymerase chain reaction. Lett. Appl. Microbiol. **24**, 351-354. (10.1046/j.1472-765X.1997.00066.x) 9172441

[4] Durand GA, Raoult D, Dubourg G. 2019 Antibiotic discovery: history, methods and perspectives. Int. J. Antimicrob. Agents **53**, 371-382. (10.1016/j.ijantimicag.2018.11.010) 30472287

[5] Silver LL. 2011 Challenges of antibacterial discovery. Clin. Microbiol. Rev. **24**, 71-109. (10.1128/CMR.00030-10) 21233508 PMC3021209

[6] Piddock LJV. 2012 The crisis of no new antibiotics—what is the way forward? Lancet Infect. Dis. **12**, 249-253. (10.1016/S1473-3099(11)70316-4) 22101066

[7] Murray CJL et al. 2022 Global burden of bacterial antimicrobial resistance in 2019: a systematic analysis. Lancet **399**, 629-655. (10.1016/S0140-6736(21)02724-0) 35065702 PMC8841637

[8] Mestrovic T et al. 2022 The burden of bacterial antimicrobial resistance in the WHO European region in 2019: a cross-country systematic analysis. Lancet Public Health **7**, e897-e913. (10.1016/S2468-2667(22)00225-0) 36244350 PMC9630253

[9] Pulingam T, Parumasivam T, Gazzali AM, Sulaiman AM, Chee JY, Lakshmanan M, Chin CF, Sudesh K. 2022 Antimicrobial resistance: prevalence, economic burden, mechanisms of resistance and strategies to overcome. Eur. J. Pharm. Sci. **170**, 106103. (10.1016/j.ejps.2021.106103) 34936936

[10] D'Andrea MM, Fraziano M, Thaller MC, Rossolini GM. 2019 The urgent need for novel antimicrobial agents and strategies to fight antibiotic resistance. Antibiotics **8**, 254. (10.3390/antibiotics8040254) 31817707 PMC6963704

[11] Tacconelli E et al. 2018 Discovery, research, and development of new antibiotics: the WHO priority list of antibiotic-resistant bacteria and tuberculosis. Lancet Infect. Dis. **18**, 318-327. (10.1016/S1473-3099(17)30753-3) 29276051

[12] Pendleton JN, Gorman SP, Gilmore BF. 2013 Clinical relevance of the ESKAPE pathogens. Expert Rev. Anti-infect. Ther. **11**, 297-308. (10.1586/eri.13.12) 23458769

[13] Santajit S, Indrawattana N. 2016 Mechanisms of antimicrobial resistance in ESKAPE pathogens. BioMed Res. Int. **2016**, 2475067. (10.1155/2016/2475067) 27274985 PMC4871955

[14] Privalsky TM, Soohoo AM, Wang J, Walsh CT, Wright GD, Gordon EM, Gray NS, Khosla C. 2021 Prospects for antibacterial discovery and development. J. Am. Chem. Soc. **143**, 21 127-21 142. (10.1021/jacs.1c10200) PMC8855840 34860516

[15] Miethke M et al. 2021 Towards the sustainable discovery and development of new antibiotics. Nat. Rev. Chem. **5**, 726-749. (10.1038/s41570-021-00313-1) 34426795 PMC8374425

[16] Alam MM et al. 2019 Design, synthesis and cytotoxicity of chimeric erlotinib-alkylphospholipid hybrids. Bioorg. Chem. **84**, 51-62. (10.1016/j.bioorg.2018.11.021) 30481646

[17] Alam MM, Hassan AHE, Kwon YH, Lee HJ, Kim NY, Min KH, Lee S-Y, Kim D-H, Lee YS. 2018 Design, synthesis and evaluation of alkylphosphocholine-gefitinib conjugates as multitarget anticancer agents. Arch. Pharm. Res. **41**, 35-45. (10.1007/s12272-017-0977-z) 29094267

[18] Farag AK, Hassan AHE, Jeong H, Kwon Y, Choi JG, Oh MS, Park KD, Kim YK, Roh EJ. 2019 First-in-class DAPK1/CSF1R dual inhibitors: discovery of 3,5-dimethoxy-N-(4-(4-methoxyphenoxy)-2-((6-morpholinopyridin-3-yl)amino)pyrimidin-5-yl)benzamide as a potential anti-tauopathies agent. Eur. J. Med. Chem. **162**, 161-175. (10.1016/j.ejmech.2018.10.057) 30445265

[19] Farag AK, Hassan AHE, Chung K-S, Lee J-H, Gil H-S, Lee K-T, Roh EJ. 2020 Diarylurea derivatives comprising 2,4-diarylpyrimidines: discovery of novel potential anticancer agents via combined failed-ligands repurposing and molecular hybridization approaches. Bioorg. Chem. **103**, 104121. (10.1016/j.bioorg.2020.104121) 32745753

[20] Hassan AHE, Lee K-T, Lee YS. 2020 Flavone-based arylamides as potential anticancers: design, synthesis and in vitro cell-based/cell-free evaluations. Eur. J. Med. Chem. **187**, 111965. (10.1016/j.ejmech.2019.111965) 31877541

[21] Kim S-Y et al. 2022 Mosloflavone-resveratrol hybrid TMS-HDMF-5z exhibits potent in vitro and in vivo anti-inflammatory effects through NF-κB, AP-1, and JAK/STAT inactivation. Front. Pharmacol. **13**, 857789. (10.3389/fphar.2022.857789) 35529447 PMC9068937

[22] Heuston S, Begley M, Gahan CGM, Hill C. 2012 Isoprenoid biosynthesis in bacterial pathogens. Microbiology **158**(Pt 6), 1389-1401. (10.1099/mic.0.051599-0) 22466083

[23] Hoshino Y, Gaucher EA. 2018 On the origin of isoprenoid biosynthesis. Mol. Biol. Evol. **35**, 2185-2197. (10.1093/molbev/msy120) 29905874 PMC6107057

[24] Zhu W et al. 2013 Antibacterial drug leads targeting isoprenoid biosynthesis. Proc. Natl. Acad. Sci. USA. **110**, 123-128. (10.1073/pnas.1219899110) 23248302 PMC3538244

[25] Boeva V. 2016 Analysis of genomic sequence motifs for deciphering transcription factor binding and transcriptional regulation in eukaryotic cells. Front. Genet. **7**, 24. (10.3389/fgene.2016.00024) 26941778 PMC4763482

[26] Warman EA, Singh SS, Gubieda AG, Grainger DC. 2020 A non-canonical promoter element drives spurious transcription of horizontally acquired bacterial genes. Nucleic Acids Res. **48**, 4891-4901. (10.1093/nar/gkaa244) 32297955 PMC7229825

[27] Rajewska M, Wegrzyn K, Konieczny I. 2012 AT-rich region and repeated sequences – the essential elements of replication origins of bacterial replicons. FEMS Microbiol. Rev. **36**, 408-434. (10.1111/j.1574-6976.2011.00300.x) 22092310

[28] Farahat AA, Ismail MA, Kumar A, Wenzler T, Brun R, Paul A, Wilson WD, Boykin DW. 2018 Indole and Benzimidazole Bichalcophenes: Synthesis, DNA Binding and Antiparasitic Activity. Eur. J. Med. Chem. **143**, 1590-1596. (10.1016/j.ejmech.2017.10.056) 29126729 PMC5744864

[29] Farahat AA, Kumar A, Say M, Wenzler T, Brun R, Paul A, Wilson WD, Boykin DW. 2017 Exploration of DAPI analogues: Synthesis, antitrypanosomal activity, DNA binding and fluorescence properties. Eur. J. Med. Chem. **128**, 70-78. (10.1016/j.ejmech.2017.01.037) 28152428 PMC5341734

[30] Nefertiti ASDG et al. 2017 Anti-parasitic effect of novel amidines against Trypanosoma cruzi: phenotypic and in silico absorption, distribution, metabolism, excretion and toxicity analysis. Parasitol. Open **3**, e5. (10.1017/pao.2017.5)

[31] Zhu X et al. 2016 Synthesis and pharmacological evaluation of mono-arylimidamides as antileishmanial agents. Bioorg. Med. Chem. Lett. **26**, 2551-2556. (10.1016/j.bmcl.2016.03.082) 27048943 PMC4841789

[32] Farahat AA, Bennett-Vaughn C, Mineva EM, Kumar A, Wenzler T, Brun R, Liu Y, Wilson WD, Boykin DW. 2016 Synthesis, DNA binding and antitrypanosomal activity of benzimidazole analogues of DAPI. Bioorg. Med. Chem. Lett. **26**, 5907-5910. (10.1016/j.bmcl.2016.11.006) 27843114

[33] Simões-Silva MR et al. 2016 Phenotypic screening in vitro of novel aromatic amidines against *Trypanosoma cruzi*. Antimicrob. Agents Chemother. **60**, 4701-4707. (10.1128/AAC.01788-15) 27216059 PMC4958229

[34] Timm BL, Da Silva PB, Batista MM, Farahat AA, Kumar A, Boykin DW, Soeiro MNC. 2014 In vitro investigation of the efficacy of novel diamidines against *Trypanosoma cruzi*. Parasitology **141**, 1272-1276. (10.1017/S0031182014000407) 24735493

[35] De Araújo JS et al. 2014 In vitro and in vivo studies of the biological activity of novel arylimidamides against *Trypanosoma cruzi*. Antimicrob. Agents Chemother. **58**, 4191-4195. (10.1128/AAC.01403-13) 24590476 PMC4068551

[36] da Silva CF et al. 2012 In Vitro and in vivo investigation of the efficacy of arylimidamide DB1831 and its mesylated salt form - DB1965 - against *Trypanosoma cruzi* infection. PLoS ONE **7**, e30356. (10.1371/journal.pone.0030356) 22291940 PMC3264605

[37] Banerjee M et al. 2012 Synthesis, DNA binding and antileishmanial activity of low molecular weight bis-arylimidamides. Eur. J. Med. Chem. **55**, 449-454. (10.1016/j.ejmech.2012.06.058) 22840696 PMC3560421

[38] Reid CS, Farahat AA, Zhu X, Pandharkar T, Boykin DW, Werbovetz KA. 2012 Antileishmanial bis-arylimidamides: DB766 analogs modified in the linker region and bis-arylimidamide structure–activity relationships. Bioorg. Med. Chem. Lett. **22**, 6806-6810. (10.1016/j.bmcl.2012.06.037) 22765899

[39] Zhu X et al. 2012 Evaluation of arylimidamides DB1955 and DB1960 as candidates against visceral Leishmaniasis and Chagas' Disease: in vivo efficacy, acute toxicity, pharmacokinetics, and toxicology studies. Antimicrob. Agents Chemother. **56**, 3690-3699. (10.1128/AAC.06404-11) 22508306 PMC3393454

[40] Daliry A et al. 2011 The trypanocidal activity of amidine compounds does not correlate with their binding affinity to *Trypanosoma cruzi* kinetoplast DNA. Antimicrob. Agents Chemother. **55**, 4765-4773. (10.1128/AAC.00229-11) 21807972 PMC3186963

[41] Branowska D, Farahat AA, Kumar A, Wenzler T, Brun R, Liu Y, Wilson WD, Boykin DW. 2010 Synthesis and antiprotozoal activity of 2,5-bis[amidinoaryl]thiazoles. Bioorg. Med. Chem. **18**, 3551-3558. (10.1016/j.bmc.2010.03.058) 20403703 PMC2892117

[42] Farahat AA et al. 2010 Synthesis, DNA binding, fluorescence measurements and antiparasitic activity of DAPI related diamidines. Bioorg. Med. Chem. **18**, 557-566. (10.1016/j.bmc.2009.12.011) 20031421

[43] Rohs R, Jin X, West SM, Joshi R, Honig B, Mann RS. 2010 Origins of specificity in protein-DNA recognition. Annu. Rev. Biochem. **79**, 233-269. (10.1146/annurev-biochem-060408-091030) 20334529 PMC3285485

[44] Amorim FTM, Blanchard JS. 2017 Bacterial Branched-Chain Amino Acid Biosynthesis: Structures, Mechanisms, and Drugability. Biochemistry **56**, 5849-5865. (10.1021/acs.biochem.7b00849) 28977745 PMC5839172

[45] Wong S-H, Lonhienne TGA, Winzor DJ, Schenk G, Guddat LW. 2012 Bacterial and Plant Ketol-Acid Reductoisomerases Have Different Mechanisms of Induced Fit during the Catalytic Cycle. J. Mol. Biol. **424**, 168-179. (10.1016/j.jmb.2012.09.018) 23036858

[46] Liang Y-F, Long Z-X, Zhang Y-J, Luo C-Y, Yan L-T, Gao W-Y, Li H. 2021 The chemical mechanisms of the enzymes in the branched-chain amino acids biosynthetic pathway and their applications. Biochimie **184**, 72-87. (10.1016/j.biochi.2021.02.008) 33607240

[47] Hassan AHE et al. 2019 Repurposing mosloflavone/5,6,7-trimethoxyflavone-resveratrol hybrids: Discovery of novel p38-α MAPK inhibitors as potent interceptors of macrophage-dependent production of proinflammatory mediators. Eur. J. Med. Chem. **180**, 253-267. (10.1016/j.ejmech.2019.07.030) 31310917

[48] Hassan AHE, Phan T-N, Choi Y, Moon S, No JH, Lee YS. 2022 Design, Rational Repurposing, Synthesis, In Vitro Evaluation, Homology Modeling and In Silico Study of Sulfuretin Analogs as Potential Antileishmanial Hit Compounds. Pharmaceuticals **15**, 1058. (10.3390/ph15091058) 36145279 PMC9504330

[49] Hassan AHE et al. 2022 Positional scanning of natural product hispidol's ring-B: discovery of highly selective human monoamine oxidase-B inhibitor analogues downregulating neuroinflammation for management of neurodegenerative diseases. J. Enzyme Inhib. Med. Chem. **37**, 768-780. (10.1080/14756366.2022.2036737) 35196956 PMC8881063

[50] Hassan AHE, Phan T-N, Yoon S, Lee CJ, Jeon HR, Kim S-H, No JH, Lee YS. 2021 Pyrrolidine-based 3-deoxysphingosylphosphorylcholine analogs as possible candidates against neglected tropical diseases (NTDs): identification of hit compounds towards development of potential treatment of Leishmania donovani. J. Enzyme Inhib. Med. Chem. **36**, 1922-1930. (10.1080/14756366.2021.1969385) 34425714 PMC8386730

[51] Kang S et al. 2018 Repositioning of the antipsychotic trifluoperazine: Synthesis, biological evaluation and in silico study of trifluoperazine analogs as anti-glioblastoma agents. Eur. J. Med. Chem. **151**, 186-198. (10.1016/j.ejmech.2018.03.055) 29614416

[52] Elkamhawy A, Hassan AHE, Paik S, Sup Lee Y, Lee H-H, Shin J-S, Lee K-T, Roh EJ. 2019 EGFR inhibitors from cancer to inflammation: discovery of 4-fluoro-N-(4-(3-(trifluoromethyl)phenoxy)pyrimidin-5-yl)benzamide as a novel anti-inflammatory EGFR inhibitor. Bioorg. Chem. **86**, 112-118. (10.1016/j.bioorg.2019.01.017) 30685642

[53] Farag AK, Hassan AHE, Ahn BS, Park KD, Roh EJ. 2020 Reprofiling of pyrimidine-based DAPK1/CSF1R dual inhibitors: identification of 2,5-diamino-4-pyrimidinol derivatives as novel potential anticancer lead compounds. J. Enzyme Inhib. Med. Chem. **35**, 311-324. (10.1080/14756366.2019.1699554) 31809612 PMC6913669

[54] Zhu W et al. 2015 Antibacterial drug leads: DNA and enzyme multitargeting. J. Med. Chem. **58**, 1215-1227. (10.1021/jm501449u) 25574764 PMC4513954

[55] Opperman TJ et al. 2016 DNA targeting as a likely mechanism underlying the antibacterial activity of synthetic bis-indole antibiotics. Antimicrob. Agents Chemother. **60**, 7067-7076. (10.1128/AAC.00309-16) 27620482 PMC5118985

[56] Rahman A, O'Sullivan P, Rozas I. 2019 Recent developments in compounds acting in the DNA minor groove. Medchemcomm **10**, 26-40. (10.1039/C8MD00425K) 30774852 PMC6349057

[57] Wang BL, Li YH, Wang JG, Ma Y, Li ZM. 2008 Molecular design, synthesis and biological activities of amidines as new ketol-acid reductoisomerase inhibitors. Chin. Chem. Lett. **19**, 651-654. (10.1016/j.cclet.2008.04.009)

[58] Bayaraa T et al. 2020 Discovery, synthesis and evaluation of a ketol-acid reductoisomerase inhibitor. Chem. Eur. J. **26**, 8958-8968. (10.1002/chem.202000899) 32198779

[59] Hong JY et al. 2019 The anti-proliferative activity of the hybrid TMS-TMF-4f compound against human cervical cancer involves apoptosis mediated by STAT3 inactivation. Cancers **11**, 1927. (10.3390/cancers11121927) 31816985 PMC6966466

[60] Hassan AHE et al. 2019 Natural products hybrids: 3,5,4′-Trimethoxystilbene-5,6,7-trimethoxyflavone chimeric analogs as potential cytotoxic agents against diverse human cancer cells. Eur. J. Med. Chem. **161**, 559-580. (10.1016/j.ejmech.2018.10.062) 30396104

[61] Farahat AA et al. 2011 Exploration of larger central ring linkers in furamidine analogues: synthesis and evaluation of their DNA binding, antiparasitic and fluorescence properties. Bioorg. Med. Chem. **19**, 2156-2167. (10.1016/j.bmc.2011.02.045) 21421317

[62] Mueller M, de la Peña A, Derendorf H. 2004 Issues in pharmacokinetics and pharmacodynamics of anti-infective agents: kill curves versus MIC. Antimicrob. Agents Chemother. **48**, 369-377. (10.1128/AAC.48.2.369-377.2004) 14742182 PMC321563

[63] Rodríguez-Rojas A, Rolff J. 2022 Antimicrobial activity of cationic antimicrobial peptides against stationary phase bacteria. Front. Microbiol. **13**, 1029084. (10.3389/fmicb.2022.1029084) 36386690 PMC9641054

[64] Guo R-T et al. 2007 Bisphosphonates target multiple sites in both *cis*- and *trans*-prenyltransferases. Proc. Natl. Acad. Sci. USA. **104**, 10 022-10 027. (10.1073/pnas.0702254104) PMC1877987 17535895

[65] Chang S-Y, Ko T-P, Liang P-H, Wang AHJ. 2003 Catalytic mechanism revealed by the crystal structure of undecaprenyl pyrophosphate synthase in complex with sulfate, magnesium, and triton. J. Biol. Chem. **278**, 29 298-29 307. (10.1074/jbc.M302687200) 12756244

[66] Chang S-Y, Ko T-P, Chen APC, Wang AHJ, Liang P-H. 2004 Substrate binding mode and reaction mechanism of undecaprenyl pyrophosphate synthase deduced from crystallographic studies. Protein Sci. **13**, 971-978. (10.1110/ps.03519904) 15044730 PMC2280048

[67] Tadrowski S, Pedroso MM, Sieber V, Larrabee JA, Guddat LW, Schenk G. 2016 Metal ions play an essential catalytic role in the mechanism of ketol–acid reductoisomerase. Chem. Eur. J. **22**, 7427-7436. (10.1002/chem.201600620) 27136273

[68] Lin X et al. 2021 Discovery of a pyrimidinedione derivative with potent inhibitory activity against mycobacterium tuberculosis ketol–acid reductoisomerase. Chem. Eur. J. **27**, 3130-3141. (10.1002/chem.202004665) 33215746

[69] CLSI. 2005 Performance standards for antimicrobial susceptibility testing; fifteenth informational supplement. CLSI/NCCLS document M100-S15. Wayne, PA: Clinical and Laboratory Standards Institute/NCCLS.

[70] Smith NM, Ang A, Tan F, Macias K, James S, Sidhu J, Lenhard JR. 2021 Interaction of *Staphylococcus aureus* and *Acinetobacter baumannii* during *in vitro* β-lactam exposure. Antimicrob. Agents Chemother. **65**, e02414-20. (10.1128/AAC.02414-20) 33495215 PMC8097447

[71] Elkamhawy A, Paik S, Hassan AHE, Lee YS, Roh EJ. 2017 Hit discovery of 4-amino-N-(4-(3-(trifluoromethyl)phenoxy)pyrimidin-5-yl)benzamide: a novel EGFR inhibitor from a designed small library. Bioorg. Chem. **75**, 393-405. (10.1016/j.bioorg.2017.10.009) 29102722

[72] Hassan AHE, Park HR, Yoon YM, Kim HI, Yoo SY, Lee KW, Lee YS. 2019 Antiproliferative 3-deoxysphingomyelin analogs: design, synthesis, biological evaluation and molecular docking of pyrrolidine-based 3-deoxysphingomyelin analogs as anticancer agents. Bioorg. Chem. **84**, 444-455. (10.1016/j.bioorg.2018.11.040) 30576908

[73] Gulia K, Hassan AHE, Lenhard JR, Farahat AA. 2023 Data from: Escaping ESKAPE resistance: *in vitro* and *in silico* studies of multifunctional carbamimidoyl-tethered indoles against antibiotic-resistant bacteria. *Dryad Digital Repository*. (10.5061/dryad.c2fqz61d1) PMC10113819 37090961

